# Retraction: Exosomal long non-coding RNA UCA1 functions as growth inhibitor in esophageal cancer

**DOI:** 10.18632/aging.204366

**Published:** 2022-10-31

**Authors:** Zijiang Zhu, Huilin Wang, Yao Pang, Hongxia Hu, Hongyi Zhang, Wenhao Wang

**Affiliations:** 1 Department of Thoracic Surgery, Gansu Provincial Hospital, Lanzhou, Gansu, China

**Keywords:** esophageal cancer, exosome, lncRNA UCA1, progression, biomarker

**This article has been retracted: **Aging has completed its investigation of this paper. We found overlap between some of the transwell assay images used for **Figures 3E **and **5E** and data published by other authors. We also found that images of exosomes isolated from the medium of NEEC and EC18 cells in **Figure 4B** duplicate exosome images from different cells published in unrelated papers. The authors reply that they “are unable to provide satisfactory data in relation to these figures and were unable to support the integrity of the research.” Consequently, all authors agreed that the article should be retracted.

